# Small-scale release of non-gene drive mosquitoes in Burkina Faso: from engagement implementation to assessment, a learning journey

**DOI:** 10.1186/s12936-021-03929-2

**Published:** 2021-10-09

**Authors:** Lea Pare Toe, Nourou Barry, Anselme D. Ky, Souleymane Kekele, Wilfrid Meda, Korotimi Bayala, Mouhamed Drabo, Delphine Thizy, Abdoulaye Diabate

**Affiliations:** 1 Institut de Recherche en Sciences de la Santé, BP 545 Bobo Dioulasso, Burkina Faso; 2 Department of Life Sciences, Imperial College London, London, UK

**Keywords:** Malaria, Genetically modified mosquitoes release, Gene drive, Stakeholder engagement, Responsible research, Evaluation, Audit

## Abstract

**Background:**

Innovative tools are needed to complement the existing approach for malaria elimination. Gene drive mosquitoes are one potential new technology in the control of malaria vectors. Target Malaria is one of the research projects developing this technology, and in July 2019, the project proceeded to an important step for this evaluation pathway: the small-scale release of non-gene drive sterile male mosquitoes in a village in Burkina Faso. In addition to the entomological and laboratory work to prepare for this important milestone, significant community and stakeholder engagement work was done. The existing guidelines on gene drive mosquito provide an overall framework for such engagement work. However, they do not provide a road map on how to proceed or what benchmarks should be used to assess this work.

**Methods:**

This study provides a review of engagement activities relevant to field trials on non-gene drive genetically-modified mosquitoes as well as an assessment framework—using both qualitative and quantitative studies as well as an audit procedure. The latter was implemented to evaluate whether the release activities could proceed with the appropriate level of agreement from the community.

**Results:**

This paper shows the importance of this first phase of work to innovate and learn about engagement processes for responsible research in the field of genetic approaches for malaria vector control. The function of these assessments is crucial for the learning agenda. The assessments demonstrated ways to increase understanding and ensure effective progress with field studies and, therefore, the pathway for responsible research.

**Conclusion:**

Gene drive technology is increasingly considered as a promising approach to control vector borne diseases, in particular malaria. Stakeholders’ involvement in this research process is one of the recurring requirements in international guidance documents. With this paper Target Malaria offers an opportunity to explore the practical achievements and challenges of stakeholder engagement during early phases of a technology evaluation, and in particular how it implemented an assessment framework to learn from its experience.

## Background

After two decades of steady progress in the fight against malaria, the decrease in cases and deaths has been stalling since 2017 [1]. Many factors explain this situation, including the change in mosquito biting behaviour and the increased insecticide resistance [1–4]. As a result, many high burden countries are losing ground [1].

The World Health Organization (WHO) is currently consulting stakeholders to update its 2016–2030 Global Technical Strategy for malaria with an added focus on the "country ownership and leadership", including community participation, and the need to foster "innovation in tools and implementation approaches" [5].

As part of this effort, scientists have been researching genetic approaches that could provide a complementary tool in the fight against malaria. Among the different ideas, harnessing naturally-occurring gene drives [6] has been considered a promising approach for vector control [7]. Gene drives are heritable elements that bias inheritance in their favour, resulting in the genetic element becoming more prevalent in the population over successive generations [8]. For instance, this technology can be used to reduce the reproduction of malaria-transmitting species (such as *Anopheles gambiae*) and thus reduce the population of this vector. As such, the technology would complement the existing vector control tools, such as Long-Lasting Insecticidal Nets (LLIN) or Indoor Residual Spraying (IRS), which have been facing challenges due to mosquito resistance to insecticide and biting behaviour changes [2, 9, 10].

Target Malaria, a not-for-profit research consortium, is among the pioneering projects exploring development of gene drive mosquitoes for malaria control [11, 12]. Its mission is to “develop and share new, cost-effective and sustainable genetic technologies to modify mosquitoes and reduce malaria transmission” [11]. The project’s work is guided by the values of excellence, co-development, evidence-driven process, openness and accountability [11]. Over the years, the project has demonstrated in contained laboratory cages in Italy and the UK the feasibility of a potential population reduction impact on malaria vectors [13, 14]. Since 2012, partners from endemic countries including Burkina Faso, Mali, Uganda and more recently Ghana and Cape Verde have joined the consortium to participate in the development of the technology. Early in this project, partners decided to take a phased approach to evaluate the technology, with a gene drive mosquito strain as the ultimate phase. The intermediary phases involve using self-limiting non-gene drive mosquito strains as incremental learning technologies. The first phase involves a strain of mosquito in which the males are completely sterile [15]. The intermediary mosquitoes aim at generating knowledge: skills for the teams, data on modified mosquito behaviour, survival, experience with the regulatory process and co-development of knowledge and relationships with stakeholder groups. A phased approach has been recommended in the key guidance documents and literature about responsible gene drive research [16, 17].

In Burkina Faso, the research is carried out under the leadership of the Institut de Recherche en Sciences de la Santé (IRSS). After years of learning about the wild type population of the target vector species [18, 19], preparing the containment facility to work with a strain of genetically-modified mosquitoes [20], engaging communities about this first phase of work [21] and after obtaining the agreement from the community surrounding the insectary facility, and the permit from the national regulatory authorities in Burkina Faso, the team at IRSS imported eggs of a non-gene drive sterile male mosquito strain [15] to its containment facility in 2016. The imported mosquito line (referred to scientifically as Ag(DSM)2) was introgressed into a colony from the local background of *Anopheles coluzzii,* and the adapted genetically-modified strain Ac(DSM)2 was maintained in the laboratory [22, 23]. On July 1st, 2019, the IRSS team proceeded to a small-scale release of approximately 6400 non-gene drive sterile male mosquitoes and approximately 8500 non-genetically-modified siblings. In line with the purpose of a phased approach, this release did not aim to impact malaria transmission, but rather to build knowledge. The two principal learning objectives were (i) to estimate the daily survival rate of male mosquitoes of the sterile male strain, and (ii) to understand the nature of their dispersal in and around release village. Also, this release was envisaged to contribute to a continuous dialogue with the authorities and affected communities about genetic approaches to malaria control.

Engagement is defined by the National Academy of Sciences Engineering and Medicine (NASEM) as seeking and facilitating the sharing and exchange of knowledge, perspectives, and preferences between or among groups who often have differences in expertise, power, and values [16]. Target Malaria's engagement strategy is structured around different types of actors, as defined by the NASEM report: communities, stakeholders and the public. Communities are understood to be groups of people who live near enough to a potential field trial or release site to have a tangible and immediate interest in the project; stakeholders have professional or personal interests sufficient to justify their engagement, but may not have geographical proximity to a potential release site; the public represents groups who lack the direct connection of stakeholders and communities, but nonetheless have interests, concerns, hopes, fears and values that can contribute to a democratic decision-making process [16].

As with the technology development process, the engagement strategy is also a phased approach that builds on existing guidance and knowledge and aims at co-developing a constructive dialogue with communities and other stakeholders to foster their participation in the technology development. Each phase helps clarify values, dynamics, and factors for Target Malaria to integrate in the development of an innovative vector control tool [24]. The outcomes will strengthen the following stages of the research and adapt the stakeholder engagement model and content.

The commitment of Target Malaria to stakeholder engagement is rooted in ethical principles [25] and a recognition that the affected communities should be involved in the development of public health interventions [16, 26, 27]. As a result, the project considers that engagement is one of its pillars, together with science and regulatory compliance. Hence, the decision to proceed with the small-scale release of non-gene drive sterile male mosquitoes—is based on the project's preparedness on those three aspects: scientific readiness [28, 29], regulatory approvals and communities' readiness. This preparedness is assessed internally through a systematic and rigorous process specific to each one of these components.

This paper examines specifically engagement and community readiness. It analyses how Target Malaria established and implemented an assessment framework of communities’ involvement in and acceptability of the proposed research activities, as recommended in international guidance documents [30]. This paper has two purposes: first, to describe and conduct a critical analysis of the engagement that led up to the small-scale release of non gene drive sterile males in Burkina Faso, and second, to examine the assessment framework put in place to review the process and outcomes of this engagement before proceeding to this release.

### Social context

Target Malaria conducted the small-scale release of non-gene drive genetically-modified sterile male mosquitoes in Burkina Faso, where malaria remains a primary public health problem with more than 7,8 million cases and 14,000 deaths a year [1]. Burkina Faso is one of the ten highest malaria burden countries in the world [1]. Conventional vectors control tools, such as LLINs, IRS and improved malaria diagnostics and treatments, have not been able to bring the country close to malaria elimination [31].

The Institut de Recherche en Sciences de la Santé (IRSS) campus, where Target Malaria conducts its research, is located in Bobo-Dioulasso, the second largest city of Burkina Faso. The research facilities, including the insectary established to host the research with genetically-modified mosquitoes, are located in Bobo-Dioulasso. This area is an ancient settlement, with a diverse population aggregating various social and linguistic groups such as Bobo, Dioula, Mosse and Fulani. The level of schooling in the district is similar to that of other cities in the country, with around 20% of children attending school [32] and the main occupation of the inhabitants is public-sector service, medium-sized private-sector businesses and local trading, often informal.

As per the permit issued by the Agence National de Biosécurité (ANB), the location for the release was the village of Bana, located in the Western Burkina Faso humid savannah zone. Malaria transmission is sustained there with high vector abundance and sporozoite prevalence [18, 19]. Located approximately 20 km from Bobo-Dioulasso, Bana has about 130 compounds where the oldest family member acts as the household head. The Bobo ethnic group, who established the village as such, is referred to as "autochthonous" and is the primary social group. The village also hosts other minority groups, including Mossi and Fulani, who established themselves in the village after its creation and are considered non-autochthonous regardless of when they established themselves. The village is under the responsibility of political chiefs, traditional chiefs, administrative representatives called the village development council (VDC). Those leaders are well-established in the community and correspond to the usual local governance frameworks in the Hauts Bassins region where the project operates. The Bobo are part of acephalous groups, which do not have a centralized and highly hierarchical power system. The main occupation in the village is subsistence agriculture and small-scale commercial vegetable production.

## Methods

**Fig. 1:**
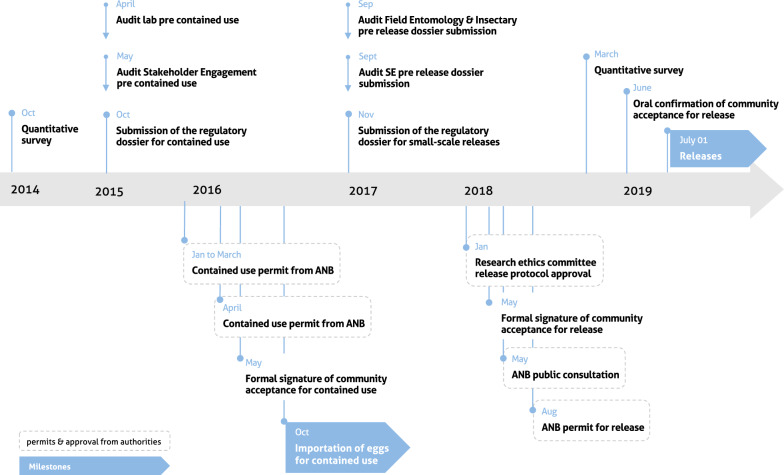
Project preparedness timeline for the small-scale release of genetically-modified sterile male mosquitoes and related regulatory milestones

Project preparedness actions for the small-scale release of genetically-modified sterile male mosquitoes included stakeholder engagement, ethnographic and quantitative studies as well as internal audits process (Fig. 1). Figure 1 describes the chronology of these preparedness activities and milestones as well as the regulatory steps. The project’s engagement and agreement process and the National Biosafety Agency’s (ANB) regulatory process (including the consultation) have independent timings. This is why for instance in the case of the contained use, the permit was issued by the ANB before the formal signature of the community acceptance, while in the case of the release it was issued after.

### Engagement methodology

Target Malaria designed its engagement strategy based on its stated values, ethical principles and recent guidance documents and literature on emerging good practices [16, 30, 33–35]. This strategy followed an iterative approach, by which the project regularly reviewed its practice according to new guidelines, changes in the study site context (including local priorities, governance landscape) and the feedback from those participating in its engagement activities.

This approach is based on several characteristics: the ability to establish a two-way dialogue and adapt to stakeholders' inputs, the focus on inclusiveness and empowerment of local communities and trust-building. The adaptation of the strategy and existing guidelines to the local context is essential in this strategy. To do so, the engagement team from IRSS conducted a longitudinal ethnographic study with the communities where Target Malaria operates, and in particular in the village of Bana, where the first release of non-gene drive genetically-modified mosquitoes was envisaged. This study was based on qualitative data collection to identify social organization and dynamics, including relationships between various groups living in the village, decision-making processes, governance structures and their legitimacy, and their perception of research and vector control. A series of 35 in-depth interviews were carried out in the local language (Dioula) by IRSS researchers during that initial phase. The sample included leaders, women, men, young people, administrative authorities and public servants working in Bana, and villagers working away from the village. This sampling did not aim at having a strict representation of village demography but rather at capturing key informants who have historical knowledge or can provide a comprehensive understanding of the groups living in Bana. In addition to these interviews, fifteen focus group discussions [36] gathering between eight and twelve people each, were also organized with specific groups such as leaders, minority ethnic groups, and youth. Project members facilitated those discussions, and participants were selected on the basis of demographic criteria (gender, age, ethnic group) and for some groups included the criteria of participation to entomological activities. The discussions aimed at clarifying, confirming and deepening project understanding about specific topics related to the proposed approach for community engagement. For instance, on the consultation model, significant time was spent with the community to discuss how their final decision would be made and recorded. The question of decision recording and the balance between the local tradition of orality—which values the “word given”—and the research need to have some form of evidence of this decision required long discussions for mutual understanding and the co-development of an acceptable solution for all. The data collection and analysis were continually done in parallel, and results were used to refine the interview and focus group guides for the subsequent interactions with the communities.

### Assessment methodologies

Early in the engagement strategy design, the project identified the need for assessing the process to ensure that the community’s decision regarding the potential release of sterile male mosquitoes would be sufficiently informed and representative. Different activities were proposed to carry out such assessment: an audit from the project on the readiness and quantitative studies of the understanding. The audit and the quantitative studies allowed the engagement team to have an evidence-based decision in this process.

#### Audit methodology

The engagement audit methodology was developed in parallel to the assessment of the field entomology readiness [29] and was preceded by a similar process for community readiness to the importation and contained use of non-gene drive sterile male mosquitoes. The first step was establishment of an internal audit checklist. This checklist aimed at assessing whether engagement activities had been carried out in compliance with the project engagement strategy, internal engagement guidelines, to evaluate whether communities and stakeholders are "ready" for the activity, and finally to consider the preparedness of the project team and its systems to manage the activity. This checklist integrates recommendations from relevant guidelines documents that are specific to area-wide vector control—including those using genetically-modified mosquitoes [16, 30, 33, 34, 37]—as well as general considerations for stakeholder engagement whether in the field of public health [38] or in other fields where stakeholder engagement has been standardized [39]. The audit checklist was reviewed and improved by Target Malaria's Ethics Advisory Committee [40]. It revolved around eight themes: 1. Identification and analysis of stakeholders, 2. Information, 3. Consultation, 4. Negotiation and partnership, 5. Complaint management, 6. Stakeholder involvement in project monitoring, 7. Feedback to stakeholders, and 8. Management functions. Table 1 articulates the audit overall criteria, objectives, evaluation criteria and evaluation methods for a sample of criteria out of the 49 criteria evaluated during the audit.

**Table 1:** Sample of the audit criteria and their evaluation for the non-gene drive sterile male mosquito release

Category	Benchmark/criteria	Target/Objective	Evaluation criteria	Evaluation method
Identification and analysis	Stakeholder identification and analysis for releases were carried out	The project identifies individuals and groups who can potentially to be directly impacted by its activities or who have a particular interest in these activities	Stakeholders are identified and analysed at the different levels: target village for release, secondary villages, regional level, around the insectary and national level	Documentation review of stakeholder mappings
Information	Stakeholders were informed and understood about the sterile male mosquitoes (what, why) and their release	Stakeholders at the target village have a sufficient level of understanding to make an informed decision Stakeholders at other levels have had access to information and opportunities of dialogue	Stakeholders at all levels are able to explain in their own words the basic characteristics of the sterile male mosquitoes At the target village level, stakeholders need to be able to explain with their own words what the release will entail, with the basic information, the key steps of the release, its preparation, and its monitoring as well as what would be expected of them. They do not express a lack of information or of responses to their questions Other stakeholders can explain basic information about the releases and feel they have had sufficient access to information and responses to their questions Stakeholders are know that this is not a vector control tool and have a basic understanding of the regulators’ role in the process	Documentation review of the meeting briefs to confirm the information provided Interviews at all levels to confirm that the information was communication and the understanding level
Consultation	The consultation process was developed in dialogue with stakeholders	Key stakeholders were invited to provide inputs for the consultation process	There is written evidence of these inputs (in the record SE) The consultation strategy developed based on dialogue with stakeholders exists and is documented	Documentation review of the stakeholder engagement records and of the consultation strategy Interviews with key stakeholders to confirm how the process of establishing the consultation took place
	The consultation was inclusive, particularly for vulnerable groups who did not have access to public debate (including women)	The consultation process provides for representation of vulnerable groups	Vulnerable groups are taken into account in the community decision-making processes, and they are satisfied with the way the consultation will include their views	Documentation review of the stakeholder engagement records and of the consultation strategy Interviews with vulnerable groups, including women
	The consultation was significant, and based on understandable and culturally appropriate information	During the consultation process, the project verifies stakeholder understanding, in particular that it is not a vector-control tool Appropriate communication tools were provided to stakeholders in advance, including an information sheet on release with appropriate information (in alignment with the ethics protocol)	Evidence of verifications of stakeholder understanding prior to the consultation Communication tools are adapted to the stakeholders’ cultural preferences and context (including the literacy level) The information provided to the stakeholders includes at least the details present in the information sheet	Document review of communication tools and of engagement records Interviews with stakeholders
Complaints management	Complaint management mechanism in place	The complaint management system(s) are in place at the different levels and able to capture and address stakeholders’ concerns, feedback and complaints	Complaint management system(s) are documented for each levels and their mechanisms (committee, forms, etc. as appropriate) are in place and ready to function or already functioning	Documentation review of the complaint management system(s) Interviews with key stakeholders at various levels
	Stakeholders are informed about the complaints mechanism and know how to file a complaint and how it is dealt with	Stakeholders at different levels were informed about this mechanism	Informed stakeholders are able to say where they can express a complaint, and how it will be dealt with Communication tools are available and distributed / displayed / disseminated to ensure knowledge of this mechanism	Documentation review of the communication tools on the complaint mechanism Interviews with key stakeholders at various levels
Involvement of stakeholders in project monitoring	A discussion is underway with stakeholders on how to involve them in monitoring the release	Information on the possible involvement in the monitoring of the release was given to the stakeholders of the target village Stakeholders in the target village were able to make an informed decision about this participation	A discussion was initiated with the field entomology and insectarium teams to identify how stakeholder representatives could participate in the monitoring of releases Stakeholders understood what the release activities will entail and were able to identify which steps/activities are of more interest to them and whether and how they would like to be involved	Interviews with team members and with key stakeholders
Feedback to stakeholders	A report on activities on contained work with the sterile male was done in an appropriate manner to the stakeholders	A feedback activity on contained use with the sterile male mosquito was done appropriately all levels	Activities were organized for stakeholders at each level according to their levels of interest, engagement preferences, and understanding levels Stakeholders were able to ask questions and get responses during these sessions with team members	Documentation review of engagement records Interviews with key stakeholders
Management functions	The ethics protocol on releases was approved by the competent authorities	An ethics protocol for the release and including the engagement activities was drafted, submitted and approved and the team is ready to implement it	A draft ethics protocol for the release of the sterile male mosquitoes was prepared in accordance with the project process and with the inputs of the different teams and was submitted to the competent authorities The final protocol received a documented agreement from the competent ethical authorities The release and engagement protocol is in accordance with this protocol accepted by the ethics committee and a checklist is in place	Documentation review of the ethics protocol

The audit was carried out in September 2017 by an internal project team including the global engagement manager from Target Malaria, an engagement team member from Mali Target Malaria team and an engagement expert external to the project. The composition of the audit team reflects the dual objective of this process: on one side the compliance/accountability purpose—to ensure that the engagement activities are aligned with the project's standard before proceeding to a critical milestone—and on the other side, the learning purpose—to ensure that audits can lead to improvement of the engagement strategy and approach. The external expert was selected based on a set of criteria: an established track record on social performance assessment and stakeholder participation, knowledge of the West African socio-cultural landscape, fluency in the French language.

The audit activities included: direct observation of field activities (including community meetings), interviews (individual and group) of staff, individual meeting and focus groups with community members and stakeholders, and document review. The audit team was introduced to the interviewees by the stakeholder engagement team who explained the audit objectives. The auditors stressed the confidentiality nature of the discussions taking place during the audit. The translation of meetings that took place in dioula (the local language) was done by the Malian auditor who understood and spoke dioula. While most activities took place at the community level where the release would take place or around the insectary where the mosquitoes to be released were produced, the audit also ensured that other levels (regional and national) were part of the process. This aligns with the project's engagement strategy that focuses on directly affected communities but also includes stakeholders at other levels who might not be directly affected by the activity but are nonetheless informed and consulted in the process.

The information from those audit activities was assessed in line with the audit checklist and a risk-based approach. For each indicator, the audit team determined whether the criteria were fulfilled or whether improvements were needed. If compliance was not fully achieved, the auditors qualified that gap as minor or major. The auditors provided recommendations on filling those gaps with a degree of priority from low to high for their implementation. Those conclusions and recommendations were shared first in a meeting on the last day of the audit with all the leadership members of Target Malaria Burkina Faso, and then through a written report with the whole Burkina Faso team and with the global project leadership.

Follow-up activities were organized to check whether the recommendations were implemented, with a combination of documentary review and new field interviews and observations. The "readiness" from a project perspective was only declared after the significant gaps were fulfilled.

#### Quantitative verification studies

**Fig. 2:**
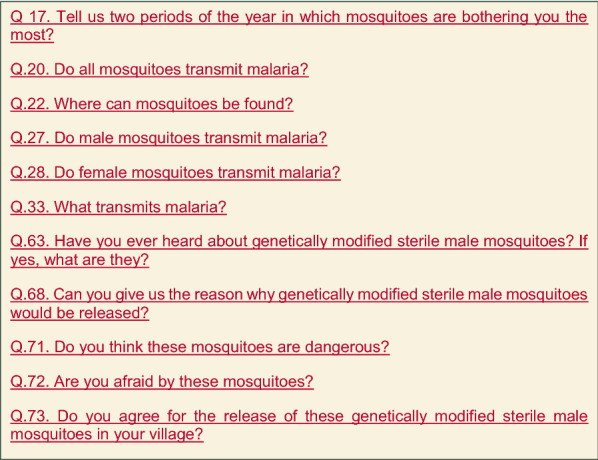
Example of Target Malaria stakeholder engagement visuals and associated messages

In addition to the audit, the IRSS engagement team also frequently carried out more quantitative studies to evaluate the level of knowledge and understanding in the Bana community. Quantitative studies were conducted in 2014, at the beginning of engagement activities and 2019, four months prior to the release, to analyse community baseline knowledge and determine the impact of engagement in knowledge-building. Surveys were focused on the following themes: sociodemographic information about the study population; their knowledge of mosquitoes and the link between malaria and mosquitoes; their understanding of genetically-modified mosquitoes; and finally, the community understanding and agreement for the small-scale release of the non-gene drive sterile male mosquitoes. All the 130 households of Bana were included in the studies, and the primary selection criterion was the role of household head. When the head was not present, another member of the household, over 18 years old (usually a spouse or a son or daughter) was interviewed. The same questionnaire (Fig. 2) was used for both surveys and it was administered to all the 130 households orally, in Dioula language. The interviewees were selected following the same method for both surveys. The questions were open-ended and the interviewer was coding them based on the responses provided by interviewees. Figure 2 provides an abstract of this questionnaire that included 77 questions. Descriptive statistics and proportional hypothesis testing were used to describe the study population and to assess its representativeness. Multivariate factorial analysis and cluster analysis then permitted the classification of different population segments according to knowledge and acceptance of genetically-modified mosquitoes and their release. Finally, a discriminant analysis model was used to measure the effect of the different factors on the level of understanding and acceptance.

## Results

### Stakeholder engagement prior to release of non-gene drive sterile male mosquitoes

#### Stakeholder identification and prioritization

Target Malaria’s engagement strategy recognizes that there is a need to engage populations that are affected to various degrees by the research activities, as the NASEM definitions allude to: communities where research is taking place and thus directly affecting the population, stakeholders who are not directly affected but who might nonetheless have an interest in the activity and require consultation, and then the public at large [16]. Taking into account this diversity of interests, the engagement strategy was specifically tailored to address the interests of each identified group. This led to a diversification of the aims of engagement, along the participation spectrum starting from information all the way to empowerment [41, 42]. This diversity is also reflected in the frequency of engagements.

Target Malaria’s commitment is that no activities should take place without the informed agreement from directly affected populations [43]. The engagement strategy was inclusive but prioritized groups directly affected by research activities, including field entomological studies, and ultimately the small-scale release of the non-gene drive genetically-modified mosquitoes. Engagement at that community level was intensive, up to weekly meetings during critical periods (prior to a new field entomology activity, including the release). As such the community living in Bana, where the mosquito collections for the characterisation of mosquito population took place, was at the heart of the engagement activities, because this community would ultimately have to make an informed decision on whether or not to allow the release in their village. Those engagements were very varied in their format, from individual meetings, to community-wide open meetings, through the use of communication tools such as theatre play or visuals.

Beyond this community, other stakeholders who could have a professional or a personal interest for the research activities were identified and engaged primarily for information purposes, but also in some cases for consultation or purely for transparency purposes. For instance, in the public sector, several stakeholders who did not have a direct role in this research oversight were engaged, such as governmental authorities, administrations from various ministries, members of parliament, governmental advisory bodies, and regional authorities to which the study sites belong. Similarly, civil society, including advocacy groups opposing biotechnologies, was considered as an important stakeholder to engage, consult and involve in the research. Openness and accountability were driving values for this engagement, but the aim of collecting their feedback for integrating into approach was also a critical motivation. The interactions with these various stakeholders were regular, up to monthly meetings towards the release period. The broader public was engaged in a second stage and with a lower frequency, through media channels, and more specifically by radio and newspaper. Information was issued via press releases, media outreach sources, and interviews carried out in the official as well as local languages.

### Building a common understanding

#### Understanding the local community dynamics and governance

Respect requires taking community values into account in the dialogue process. Before attempting to communicate anything about the science, the stakeholder engagement team spent time looking in detail at the nature of the local communities: their social organization, customs and traditions, their power structures, legitimacy issues and values.

Findings gained from these investigations highlighted entry points in the village, defined official and non-official power structures, identified dominant and minority social groups and also the quality of relationship between village leaders and administrative authorities under whose jurisdiction the village falls. Knowledge generated was used to support the engagement strategy. Identification of various centres of power has supported stakeholder mapping with the objective of minimizing the risk of missing certain groups. The analysis of the power structure has contributed to broadening engagement activities in terms of different categories of stakeholders living in the village, including minority groups. The understanding of these various aspects of social organization had an important bearing on legitimization in collective matters and was used as a foundation for co-developing a model for community consultation at the next stage.

#### Knowledge building

Early in its engagement strategy, the Target Malaria team in Burkina Faso established the importance of strengthening this knowledge as a foundation for subsequent dialogue about the project’s proposed intervention. While information and knowledge-building do not summarize the aim of Target Malaria’s engagement strategy, they are its cornerstone. As stated in the WHO Guidance Framework scientists should “ensure that the project is well understood”. Engagement “creates opportunities for knowledge exchange and mutual learning” [30] even if that dialogue does not necessarily lead to an adoption of proposed research. Knowledge engagement is crucial to create a dialogue, ensure that communities, where the research takes place, can make an informed decision about proposed activities and furthermore take part in research development [25].

Target Malaria’s approach, for a malaria vector control complementary tool, requires an understanding of the role of mosquitoes in malaria transmission, the differentiation between different species, male and female mosquitoes as well as the role of swarms in mosquito mating. It also requires community members to understand the basis of inheritance. Finally to make an informed decision, the communities also need to understand the protocol proposed and its implications.

**Fig. 3:**
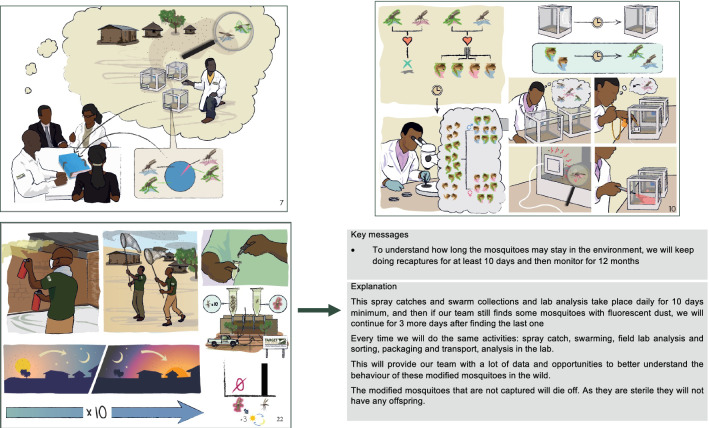
Excerpt of the questionnaire used during the quantitative survey (English translation from the French version)

The process to build this understanding is a long one, articulated around various activities from individual meetings with leaders from identified social categories of the communities, to large group meetings. During this phase of knowledge building, various communication tools are used. The first one was to co-develop with the release community a common glossary and understanding of terminology, for concepts that might not have a direct translation into the local language [44]. In addition to this, visuals were developed and used to share knowledge and get the community members to engage with this knowledge and new information. These visuals (Fig. 3) had been piloted in the preparation for the contained use in the community around the insectary and had showed to be efficient in getting the community interested and engaged with the information. These visuals (that included 22 different slides) were presented by team members during series of individual or group meetings using A0 printed versions. These led to discussions with community members that helped building the understanding. Prior to the release and following requests from the communities to diversity the communication tools, theatre play was also used to strengthen the understanding and foster dialogue around the release with a broader audience from the community. In contrast, at the regional and national levels, the communication tools used were more of a written form, such as brochures or booklets. This difference is largely due to the literacy level differences between these two groups of stakeholders. The importance of ensuring that the local community who would be potentially more directly affected by the project activities would have sufficient information to make a decision also drove some of these choices, as the visuals and theatre play allowed to provide more in-depth details on the protocol for instance.

A comparative analysis of the Bana community’s understanding of mosquitoes and malaria (Table 2) shows the improvement about the knowledge of the community on mosquito reproduction (knowledge of swarms and role of swarms in mating) and a significant reduction of common misperceptions about mosquitoes (Table 2).

**Table 2:** Comparative picture of community knowledge on mosquito and malaria in 2014 and 2019

Community knowledge about mosquitoes and malaria	2014 (%) (N = 179)	2019 (%) (N = 149)	Significant
Accurate statements			
Existence of many kinds of mosquitoes	68.72	92.62	*p* < *0,01*
Female mosquitoes transmit malaria	94.41	96.64	*p* > *0,1*
Knowledge of swarms	4.47	53.02	*p* < *0,01*
Role of swarms in mosquito-mating	3.36	30.20	*p* < *0,01*
Inaccurate statements			
All mosquitoes transmit malaria	76.3	16.11	*p* < *0,01*
Male mosquitoes transmit malaria	77.65	10.07	*p* < *0,01*
Seasonal fruits transmit malaria	13.94	0.94	*p* < *0,01*
Fatty foods transmit malaria	34.55	1.34	*p* < *0,01*

### Community decision-making process

**Fig. 4:**
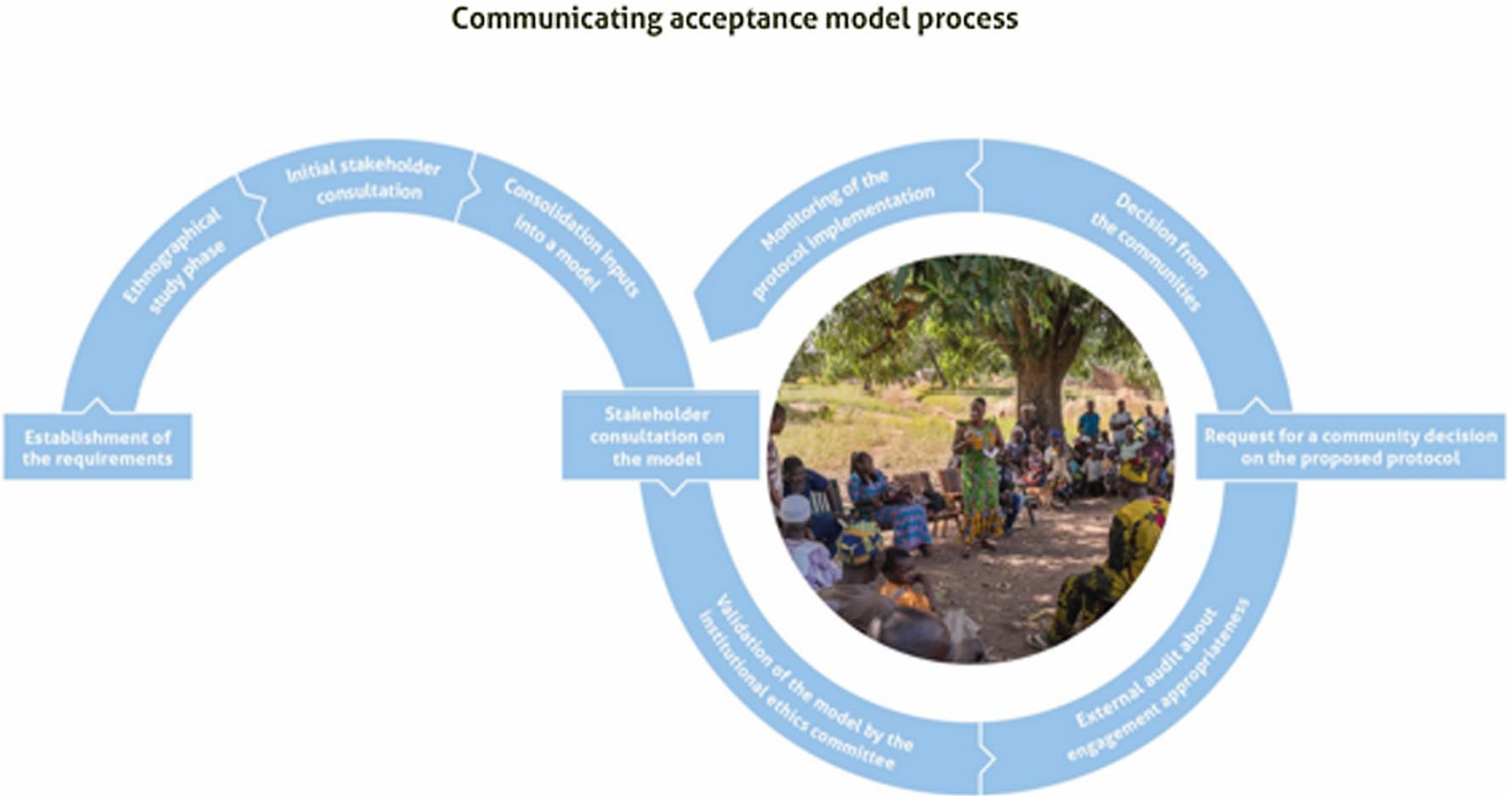
Community acceptance model

The acceptance model for release was co-developed by the research team and community members (Fig. 4). The objective was to ensure that the community could take an informed decision about the release and the associated activities as described in the research protocol. This co-development model went through different phases, including developing the acceptance model, selection of community representatives authorized to speak on behalf of the community, validation of the representatives committee by the community, design of the acceptance form, and the traceability of the agreement. This model built on research done to understand community dynamics and governance and a long dialogue with various community components. This dialogue led to the identification of community representatives. Those representatives emerged from ethnographic study that included individual and group discussions with the various community members (women, men, youth, autochtonous and non-autochtonous groups, representatives of family compounds, religious and traditional authorities), as well as observation of community dynamics. An initial list of representatives that would be allowed and legitimate to express the community decision was identified. This initial list was further cross-checked with all the village components, including the minorities and vulnerable groups. And finally, once the list was confirmed through this process, a community-wide meeting took place where the project presented that list back to the whole community for confirmation. The model, including the information sheet and acceptance form, was reviewed by the IRSS Research Ethics Committee (REC). After the REC’s approval, the project team asked community representatives for their formal decision which led to their signature on the acceptance form (Fig. 4). The community acceptance form was a four pages document, composed of two sections: an information sheet and a signature sheet. The information sheet included the key information about the study: introduction to the project context, project steps, information about the sterile male mosquitoes, objectives of the release, compliance with regulatory requirements, ethical considerations, benefits, team in charge of the protocol, confidentiality of the information, expected results and the contact details for the team and for the institutional ethics committee. The signature sheet summarized the study in a few sentences and included the names of the representatives and the fact they were expressing the community decision and had a space for the representatives’ signatures and the project team’s signature. Four representatives—whose legitimacy had been confirmed during the process—signed on behalf of the village: the chief, a councillor and two members of the VCD bureau. A project team member who was working directly in the village also signed the form.

### Fostering a continuous dialogue with stakeholders

#### Tailoring engagement to different stakeholders

Engagement with stakeholders has been guided by principles of inclusiveness, transparency, step-by-step and incremental process. The strategy tailored the approach to stakeholder interests, creating groups of stakeholders based on their interest in the research activities. Criteria ranged from having an interest in the field of activity (distant or close to the research material, such as a focus on malaria, or genetically-modified organisms); having an area of expertise (governmental-public–private-civil society); being part of a power structure (decentralized government, traditional authorities and religious authorities); or participating in a wider public dialogue around genetically-modified organisms.

Since every category included several individuals and groups, engagement focused as a priority on those who met most criteria relating to the category, the ability to provide information to other group members of the category, to influence research activities positively or negatively, or in need of information.

#### Tailoring engagement to different stakeholders

To sustain dialogue with groups of stakeholders, a system of contact persons was put in place. The research team had encouraged stakeholder groups to nominate representatives to act as focal points for the Target Malaria research. Each representative would receive information from the research team on how research was progressing, share this with colleagues and in return, collect any concerns or questions and report these back to the research team. They were also responsible for helping with organizing larger meetings.

### Engagement process and outcomes prior to the release of non-gene drive sterile male mosquitoes

#### Stakeholder identification and analysis process

Part of the audit checklist looked at the process and outcomes of the stakeholder identification and analysis process done by the project team. This was an important criterion as this process is the first step of an engagement strategy [45] and a critical one from an ethical perspective. It ensures that engagement activities are inclusive in particular for vulnerable groups, taking into consideration social determinants of health and community dynamics to prevent an accentuation of marginalization or conflicts [27]. This identification and analysis process is also important from an effectiveness perspective as it aims at identifying and engaging those who will be important in relaying opinion within the community and ensuring that any decision-making processes used by the project is legitimate.

The audit demonstrated an extensive mapping and understanding of stakeholders at all the levels (villages, insectary neighbourhood, regional and national). At the village level, this understanding was gained from ethnographic studies that highlighted “gate-keepers” in the community, official and non-official power structures, dominant and minority social groups and also the quality of relationship between village leaders and administrative authorities. The audit revealed that this process was continuous and that stakeholder maps were constantly updated and that it contributed to broadening engagement activities in terms of different categories of stakeholders living in the village, including minority groups. However, it showed some gaps in the analysis of power dynamics and relationships between different social levels and how this could impact the decision-making processes.

#### Assessment of the understanding

Along the engagement process, informal assessment of the information understanding were carried out, based on feedbacks from stakeholders and conversations between engagement team and community members. This allowed the project team to adapt its information strategy, reinforcing messages that were unclear, looking for other communication tools when needed.

During the audit, respondents commended the IRSS efforts to use different methods to build understanding throughout the years, highlighting the importance of this early engagement that had started more than five years before the audit. The audit process also revealed the critical role that community casual workers and study participants played in building community knowledge about mosquitoes as a malaria vector, Target Malaria, and the proposed activities. Over the years, a group of forty-three members of the village youth was trained to participate in field entomology data collection activities [46] and the audit showed that they participated in sharing knowledge with the rest of the community. The implementation of several mark-release-recapture studies with wild-type mosquitoes in previous years [18] helped raising the community understanding for the non sterile male mosquitoes release, that followed a very analogous protocol.

The project had set some targets for the community understanding, which had been reviewed by the ethics advisory committee. These targets were to ensure that the community understood the key characteristics of the mosquitoes to be released (in particular the fact they were genetically-modified and that the males were sterile), the key aspects of the release and recapture protocol (how the release was going to take place, the monitoring activities following up and their potential duration) as well as the fact that this was not a final technology that would impact malaria transmission, and therefore the absence of benefits in terms of public health other than a contribution for future research phases. The understanding was assessed by asking interviewees to explain what they understood this study to be and by benchmarking these responses to the target level of understanding.

The audit highlighted an overall good understanding of essential information at the community level in Bana and across various groups (men, women, leaders, youths), but noted that the same understanding level had not been yet acquired at the secondary field site of Souroukoudingan, regional or national level. While this could be explained by the fact that the Bana community was the one that would be asked to make an informed decision about the release, the recommendation was to increase the knowledge level in other locations, for instance, by adapting some of the engagement tools that the respondents had praised in Bana. The subsequent follow-up verifications of the audit recommendations’ implementation confirmed the positive results from increased information activities at other levels. Only after this step of verification was the formal decision asked from the community representatives. This sequence—ensuring first the community understanding before asking for a formal decision – was established to make sure that the decision would be sufficiently informed.

Finally, the formal quantitative study was done in 2019 allow to assess the affected community’s understanding of the release. This new quantitative study was done early 2019 as the audit that checked the understanding level had taken place in 2017 and the team wanted to confirm that the community members still had a good understanding of the protocol prior to asking their representatives verbally to confirm the release approval they had given in 2018. The quantitative study found that 56% (76/126) interviewees were able to describe the release protocol involving male mosquitoes that would not have progeny after mating with wild female mosquitoes. The remaining interviewees understood the mosquitoes were genetically-modified. Responses from 93% (138/148) of interviewees stated that the genetically-modified sterile male mosquito was not dangerous, and 96% (143/148) were in agreement with the research implementation in their village. This survey was used as an additional verification tool for the project to ensure that the agreement provided by the representatives of the village was supported by a majority of the population, confirming the decision from their representatives.

#### Assessment of the consultation

When the audit checklist for release of the non-gene drive sterile male mosquitoes was being designed, a fundamental question was raised: what are the appropriate criteria to assess the consultation? The question of whether to focus on the consultation outcome (e.g., the community agreement or individual consent for specific activities) or on the consultation process is a recurring one [47]. The decision was to focus on the consultation process as this audit was taking place prior to submission of the regulatory application and thus at a time before the release but highlighting the necessary verifications that would be needed closer to release to ensure that the release would only happen if appropriate agreement had been given by community members.

Two main criteria were assessed: whether 1. “The consultation process was developed in dialogue with the community”, and 2. “Key stakeholders understand and accept the consultation process”. The first criterion corresponds to the project’s strategy to co-develop the consultation and decision-making process with affected communities, also safeguarding vulnerable populations by ensuring that they were part of this co-development. The second criterion considers the broader legitimacy of the consultation model and is complementary to the IRSS ethics committee review of that model. The auditors approached legitimacy from two angles: whether stakeholders who are not directly affected understand and accept the idea that the decision on potential release would be made by the directly affected community and the regulatory authorities, and whether those stakeholders recognized that the model co-developed with the community was appropriate.

When looking at the process by which the consultation model was developed with the community, it appeared that understanding the social organization through ethnographic studies had been critical for co-development of this model. In fact, the consultation and decision-making process had been informed by community governance analysis. This process allowed the Target Malaria team to ensure that representatives proposed by community members were legitimate for the whole community, including vulnerable groups. That process went through several phases, including developing the acceptance model, selection of community representatives able to speak on behalf of the community, validation of the committee of representatives by the community, design of the acceptance form, and the traceability of the agreement. The audit team carried-out several interviews with groups usually marginalized in decision-making—such as women, youth and ethnic minority—to confirm that the representatives selected to express the decision on behalf of the community were considered legitimate and whether those individuals and groups felt that their perspective would be taken into account. Those interviews confirmed the results of the ethnographic studies and the dialogue on the consultation model, and their support for the delegates selected to represent the community. Similarly, the quantitative study showed that when asked individually about their perspective on the potential release, there was no significant difference between the opinion from those groups and the opinion expressed from majority groups or from the representatives of the community that they had selected.

## Discussion

### Engaging different level of stakeholders

#### Challenge to establish the level of details and information for each group

One of the decisive questions in the engagement process and its assessment is what constitutes an "informed decision" when considering release of a non-gene drive genetically-modified mosquito. The current literature about the informed decision for genetic approaches to vector control [27, 34, 35, 48, 49] does not answer the question about the minimum information provided before a community is asked to make a decision. Guidance for clinical trial participant information sheet [50] can be considered a good proxy for the minimum information to provide can be from an ethical and public health perspective, even though the studies with modified mosquitoes are not considered as clinical trials involving human subjects [30, 48]. IRSS developed an information sheet that would accompany the community agreement, and the institutional ethics committee approved the content of that information sheet as part of the release protocol. This content included basic information about the project, the mosquito strain to be released, the release and monitoring protocol, potential benefits and risks, community participation, confidentiality and freedom to participate or not with no consequences on future access to the study outcomes. This was then considered the minimal information that the community should understand before being asked to make a decision, and thus was the basis for the audit and subsequent verifications of understanding. In addition to this information, the project was committed to responding to any other question that community members or other stakeholders might have about its proposed study, including molecular construct details should that be a topic of interest. During the audit, respondents showed that potential risks and benefits and practical considerations for the study were their central considerations rather than the mechanism of genetic modification.

During the engagement period prior to the consultation and community decision-making, it was made clear that there would be two levels of decision: a community decision related to the genetically-modified mosquito release and to the overall study activities, and individual decisions related to mosquito collection activities taking place in houses (insecticide spray catch). As for the previous studies of marked release recapture done with wild-type mosquitoes, the project explained the right not to participate to the study—for the community, and for individuals who would not wish to have mosquito collection in their house. The question of individual freedom to participate in area-wide activities such as the mosquito release was not raised by community members, neither for wild-type mosquito releases nor for genetically-modified mosquito release. The results of the ethnographic study and dialogue seem to indicate that in the communities of Bana and Souroukoudingan, the decision of the group through its representatives took precedence over individual decisions.

The question about the level of information and understanding from stakeholders and the broader public can also be asked. The guidelines mention the requirement to share information and engage with this larger group, but does not set clear standards about the level of information needed [16, 17, 27]. This poses a clear challenge for research projects as they are left without clear guidelines on their obligations, but faced with societal expectations about developing a public understanding and dialogue on their research as part of the responsible research principles [51, 52]. In the “Core commitments for field trials of gene drive organisms”, authors highlighted the importance of having a fair partnership with stakeholders and regulators in the potential countries of future gene drive releases, to identify the appropriate level of accountability, multi-directional learning [53]. This corresponds to a very case-by-case approach, taking into consideration the local or national specificities and preferences, and basing the standards in a mutually agreed framework between the research projects and their stakeholders.

#### Focusing on the directly affected communities for an informed decision

Respecting research participants’ autonomy is recognized as a manifestation of their right in the research process, and informed consent as one of the most important safeguards for respecting their autonomy [54–58]. If community acceptance and not individual informed consent is recognized as the most appropriate model to consider the community voice in genetic modification research, including gene drive [55–57], the question of community understanding of the research for informed acceptance remains a challenge.

A community’s autonomy in making its own decisions should be supported by an empowerment process and be accompanied by appropriate safeguards (such as having procedures for ensuring understanding and an appropriate ethics committee’s oversight). The affected community has a role to play in the progress of research: this is something that is widely acknowledged [53], but it comes with a lot of challenges in terms of ensuring understanding and acceptance, and this deserves our serious consideration. Any form of responsible research needs to engage with the issue. Furthermore, active collaboration between a research team and communities and feedback from the latter can also influence the process of the research.

Engaging an affected community at an early stage in the research process provides a significant opportunity for increasing their level of understanding and obtaining an informed decision. The critical question can be how to ensure their understanding. The early engagement allowed the project to build the process for an informed decision through various interactions, integrating community’s feedback into the process. The training of community members for entomology collection, the activities (such as the insectary facility visits) contributed to demystifying the research and ensuring a real two-way dialogue based on the community’s understanding of the research.

### Assessing the engagement work before proceeding to a key activity

#### The need to assess

Existing guidelines and literature mention the question of engagement evaluation. The NASEM report mentions the question of evaluation in one of its recommendations: *“Researchers, funders, and policy makers should develop and implement plans to evaluate engagement activities related to gene drive research. When possible, these evaluations should be published in the scholarly literature or otherwise made available as part of a shared repository of knowledge”* [16]. While Thizy et al*.* [35] refers to the need to have “independent evaluations” and for the “outcomes and lessons learned [from these evaluations] to inform […] current practices as well as future engagement plans invite to improve their effectiveness”. However, it is clear from these texts and the absence of this question in other key guidelines on engagement and genetically-modified mosquitoes, that evaluation is only seen as a learning opportunity that takes place after the event*.* Assessments of the engagement process and outcomes are not described as part of the process required before proceeding to a specific activity or asking for informed consent.

This gap can potentially be explained by the existence of other accountability mechanisms, whether the oversight role of the Research Ethics Committee that ensures that research is implemented according to the protocol approved, and the regulatory authorities that often integrate public consultation as part of their biosafety process, for instance in implementation of Article 23 of the Cartagena Protocol on Biosafety [59].

#### Target malaria’s approach to this assessment

Audits and assessments can have two different objectives: compliance checking for accountability, and learning. The tools and approach were designed with these two objectives in mind. On one side, this involves the compliance/accountability purpose—ensuring that engagement activities are aligned with the project standards before proceeding to a key milestone—and on the other side, the learning purpose—ensuring that audits and studies can lead to improvement of the engagement strategy and approach.

Decisions regarding project activities would therefore proceed based on the milestone set, if deemed appropriate, while the new learning feeds into engagement for the next phase or other sites. This criterion was considered an internal pre-requisite, similar to the regulatory permits and the scientific activities and teams [29]. This internal high standard in terms of engagement and community acceptance is a direct response to the project's values of excellence, evidence-based decision, co-development and accountability [11]; as well as a management measure for project risk related to the first release of a genetically-modified mosquito in an African country.

The audit process does create tension between values of accountability and co-development. The accountability value requires that some compliance measurement be done, including by experts external to the local team directly responsible for engagement with communities and stakeholders. At the same time, the value of co-development implies that the community is empowered enough to make its own decisions and should be trusted in making such a decision. At the project level, co-development also requires that the national partner (in this case, IRSS) leads the work and is best placed to judge its preparedness level.

#### Limitations and challenges

Another important limit of the audit and other verification studies as accountability mechanisms is their inner nature. The audit team structure—with several auditors, external to the Burkina Faso team, and with the presence of an external expert—serves an internal project quality objective. These processes demonstrate that Target Malaria builds in control mechanisms to ensure that the engagement work is done in a rigorous manner, in a way similar to quality assurance in laboratory work or in field entomological work. However, they are not intended to guarantee that the community deliberation and decision respond to the ethical standards and existing guidance on the release of genetically-modified mosquitoes. That is precisely the role of the research ethics committee that oversees the implementation of the research protocol and ensures that participants and communities involved in a field entomology study are involved in “in ways that uphold human rights, and respect, protect, and are fair to study participants and the communities in which the research is conducted” [60]. The audit and verification processes are an internal tool to ensure that the project is ready to comply with the ethical obligations set by its research protocol.

When analysing concerns from advocacy groups or individuals who criticized the engagement process around this release and, in particular, the concern over communities being used as “guinea pigs” [61], there is no reference to any oversight mechanism of engagement, individual consent or community acceptance processes. This could denote either an absence of knowledge about existing oversight mechanisms—whether the research ethics committee or the national biosafety authorities' public consultation process—or mistrust of those mechanisms. Both processes—the research ethical approval and the regulatory public consultation—are relatively close to the general public eye and do not include a public reporting of their findings before implementing the study. This gap between existing oversight mechanisms, applied by a project, and on the other side, a particular public perception, indicates a need for greater public descriptions of the internal processes, their purpose and how they can link to external, public consultation leading to community acceptance.

This gap, amongst others, was raised during a review of Target Malaria's acceptance model carried out with a variety of experts on bioethics, stakeholder engagement and innovation governance [62]. The question of who should establish the evaluation criteria for this engagement and acceptance mechanism is as important as who carries out the assessment. This interrogates the place of experts, regulators or other institutional oversight bodies and affected community in establishing and driving the engagement and acceptance model for this technology. It also questions the impacts of self-regulation and researchers’ and other research actors’ public commitments to responsible research [53, 63] on public concerns. The drive for best practices, responsible research and fairer partnerships is present in the gene drive research community, but so far, it does not seem to influence the perception from concerned groups who express a lack of trust in regulatory and scientific processes. While there could be a temptation to call upon a "neutral third party" [64], it overlooks the question of trust needed for any oversight or monitoring mechanism.

## Conclusion

The small-scale release of non gene drive sterile male mosquitoes in the village of Bana was envisaged as a learning and capacity-strengthening activity. While this release was certainly a first in terms of scientific achievement (the first release of a non-gene drive genetically-modified insect in Africa), it was also approached as an opportunity to learn and to innovate in the field of stakeholder engagement. Aligned with its values of excellence, evidence-based and accountability, Target Malaria implemented a rigorous internal assessment process of its engagement and the acceptability level before this release. This verification complements the formal process from the research ethics committee and regulatory authorities and is another opportunity for responsible management, accountability and learning.

The stakeholder engagement process, including the assessments, was crucial to building trust and empowerment with directly affected communities and other key stakeholders. These learnings will be critical for the project's next steps of engagement with communities, stakeholders and the broader public. They demonstrate the importance of responsibility and accountability mechanisms that can provide public confidence in how the project has been engaging communities and stakeholders with the appropriate respect for their autonomy and deliberation process. Addressing this requirement for more public accountability while maintaining the project's co-development dimension, which has been at the heart of its approach and of the trust built with the community, will be the central challenge for the next phase of research.

## Data Availability

The datasets used and/or analysed during the current study are available from the corresponding author on reasonable request.
